# Assessing Health Information Seeking Behaviors Among Targeted Social Media Users Using an Infotainment Video About a Cancer Clinical Trial: Population-Based Descriptive Study

**DOI:** 10.2196/56098

**Published:** 2025-03-03

**Authors:** Jonathan Sommers, Don S Dizon, Mark A Lewis, Erik Stone, Richard Andreoli, Vida Henderson

**Affiliations:** 1 Digital Health Networks, Los Angeles, CA, United States; 2 Brown University, Providence, RI, United States; 3 Intermountain Health, Murray, UT, United States; 4 Fred Hutchinson Cancer Center, University of Washington, 1100 Fairview Ave N, Seattle, WA, 98109, United States, 1 2066676355

**Keywords:** cancer clinical trials, digital media, social media, infotainment, recruitment, education and awareness, edutainment, public engagement, cancer, lack of information, social media, health information, medical awareness, video series, public audience, low cost, research participants

## Abstract

**Background:**

The lack of information and awareness about clinical trials, as well as misconceptions about them, are major barriers to cancer clinical trial participation. Digital and social media are dominant sources of health information and offer optimal opportunities to improve public medical awareness and education by providing accurate and trustworthy health information from reliable sources. Infotainment, material intended to both entertain and inform, is an effective strategy for engaging and educating audiences that can be easily disseminated using social media and may be a novel way to improve awareness of and recruitment in clinical trials.

**Objective:**

The purpose of this study was to evaluate whether an infotainment video promoting a clinical trial, disseminated using social media, could drive health information seeking behaviors.

**Methods:**

As part of a video series, we created an infotainment video focused on the promotion of a specific cancer clinical trial. We instituted a dissemination and marketing process on Facebook to measure video engagement and health information seeking behaviors among targeted audiences who expressed interest in breast cancer research and organizations. To evaluate video engagement, we measured reach, retention, outbound clicks, and outbound click-through rate. Frequencies and descriptive statistics were used to summarize each measure.

**Results:**

The video substantially increased health information seeking behavior by increasing viewership from 1 visitor one month prior to launch to 414 outbound clicks from the video to the clinical trial web page during the 21-day social media campaign period.

**Conclusions:**

Our study shows that digital and social media tools can be tailored for specific target audiences, are scalable, and can be disseminated at low cost, making it an accessible educational, recruitment, and retention strategy focused on improving the awareness of clinical trials.

## Introduction

A total of 90% of Americans use social media [1] and over 40% of Americans watch web-based videos daily [2]. Digital media is a dominant source of health information, with 50% to 80% of internet users searching for health information on the web [3-5]. Unfortunately, misinformation and disinformation threaten the quality of health information available [6,7]. Given public interest in accessing web-based health information and the potential public reach, social media offers an optimal opportunity for public health practitioners and health care providers to improve public medical awareness and education by providing accurate and trustworthy sources of health information. However, the attention span of social media audiences is typically short. Thus, digital content has to be engaging, meaningful, and attention grabbing [8,9].

One specific area of health care that can benefit from such strategies is cancer clinical trials. The lack of information and awareness about clinical trials, coupled with significant misconceptions about them, persist as major barriers to cancer clinical trial participation [10]. Only 3% to 5% of patients with cancer participate in clinical trials and over 50% of Americans report that the lack of awareness and information are major reasons for low participation rates [11-13]. This is the case despite one study reporting that 56% of respondents preferred the internet as a source of information about clinical trials [11].

Infotainment is defined as the delivery of broadcast material intended to entertain, engage, and inform [14]. Examples of this form of media in our culture vary but can include news talk shows, podcasts, and social media influencers. Its use may be a novel way to improve awareness and alleviate misconceptions that act as barriers to clinical trials. Moreover, since many trials close due to the lack of accrual [15], infotainment delivered on social media may be a viable clinical trial recruitment strategy.

The purpose of this study was to evaluate whether a clinical trial promotional video, disseminated on social media, could effectively drive health information seeking behaviors and, as a result, increase awareness about the clinical trial. To accomplish this goal, we instituted a dissemination and marketing process to measure health information seeking behaviors among public audiences on social media.

## Methods

### Video Development and Content

A clinical trial promotional video represents an underexplored method for engaging with targeted audiences. The trial we chose to feature was Southwest Oncology Group (SWOG) S1501, “Prospective Evaluation of Carvedilol in Prevention of Cardiac Toxicity in Patients with Metastatic HER-2+ Breast Cancer, Phase III.” [16] Collaborating with a clinical trial recruitment and retention specialist from the SWOG, we selected this trial based on its relative ease of understanding and to improve accrual performance. Our research team collaborated with the SWOG Cancer Research Network, a global cancer research community that designs and conducts federally funded clinical trials; The Hope Foundation for Cancer Research, a public charity with the mission of raising and contributing funds for the treatment and prevention of cancer; and Digital Health Networks (DHN), a media production studio and streaming service that specializes in producing, marketing, and distributing health care content and collecting viewership data.

The creation of the video was informed by the World Health Organization (WHO) Strategic Communications Framework [17] and the social cognitive theory [18]. The WHO Strategic Communications Framework asserts that effective health communications should be accessible, actionable, credible and trusted, relevant, timely, and understandable [17]. The social cognitive theory asserts that learning occurs in social contexts where individuals can influence and be influenced by others and their environment [18]. Using the social cognitive theory framework in the context of the health information seeking behavior of social media users, Zhang et al [19] found that (1) information quality, social media platform quality, and user experience have a significant positive effect on emotional arousal; (2) user experience, social support, and emotional arousal have a significant positive effect on self-efficacy; and (3) emotional arousal and self-efficacy have a significant positive effect on social media users’ health information seeking behavior. To this end, we sought to develop high-quality, emotionally engaging digital media that delivers accurate information about a clinical trial, with an embedded URL hyperlink to learn more information from a credible source, on a highly used social media platform to increase awareness about the trial and motivate users to seek additional information.

To ensure the information, language, and imagery provided in the video was medically accurate, a project steering committee, who chose the clinical trial of focus, was formed, consisting of SWOG Digital Engagement committee members, study investigators, and a representative from The Hope Foundation for Cancer Research. The video concept, script, and storyline were initially developed by a research team member who is also a filmmaker; cancer survivor; and long-standing member of the SWOG’s Digital Engagement, Adolescent and Young Adult, and Patient Advocates committees. The outline, script, and questions were submitted to the project steering committee for feedback, and regular communication was maintained throughout the process in multiple feedback loops. Updates were provided during committee meetings and were presented at biannual SWOG group meetings with storyboards, a rough and final cut of the video, and initial project results. This inclusive process encouraged committee members and meeting attendees to provide feedback, ask questions, and be part of the creative filmmaking process. The steering committee viewed the video and gave the final sign off prior to launch.

**Figure 1.**
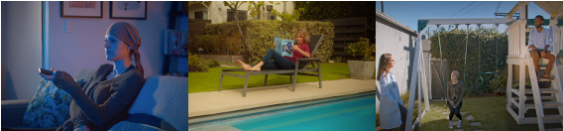
Video screenshots showing (left) the main character (Beth), (middle) a paradoxical patient with cancer, and (right) a scene with clinicians explaining the clinical trial to Beth.

To engage audiences, the video attempted to parody cliché tropes often seen in pharmaceutical commercials. In our fictional video, Beth, a more relatable portrayal of a woman who is visually worn down from cancer treatment, is watching television and flipping through channels as a highly stylized, cheery commercial for the S1501 study comes on. It is akin to pharmaceutical advertisements commonly broadcast on television. Beth, feeling the side effects of treatment, dozes off but is awakened when she, in disbelief, is interacting with the commercial’s characters and asking the physicians questions about clinical trials and specifically the S1501 study. In this narrative structure, Beth’s role is to serve as a vehicle to convey the pertinent information the study team hoped the audience would learn. In the end, Beth is brought back to her reality. Appearing cautiously interested in the trial, she presumably seeks out further information about it. Video screenshots showing the main character (Beth), a paradoxical patient with cancer, and a scene with clinicians explaining the clinical trial to Beth is provided in Figure 1. The full video is available in Multimedia Appendix 1.

### Ethical Considerations

The National Cancer Institute Cancer Prevention and Control Central Institutional Review Board reviewed the recruitment/patient education video (protocol version dated June 6, 2019; study ID: S1501) and granted approval on July 24, 2019. The expedited review was conducted in accordance with the federally defined categories of expedited review stated in 45 CFR 46.110(b)(1)(ii) and 21 CFR 56.110(b)(2). The dissemination strategy was not determined to be human subjects research.

The study protocol and study materials for video 2 were reviewed and approved by the National Cancer Institute Institutional Review Board. We are publishing the results from a marketing strategy to see how it could inform research. The data presented herein represent information collected for the purposes of social media marketing, and we did not collect individual-level data. Rather, we collected population-level data to inform the effectiveness of marketing research. Institution review board approval and informed consent were not obtained as no human subject data were collected on an individual level, no data were obtained through interaction with any individuals, and we cannot ascertain the identities of individuals to whom data the belonged. The data collection was conducted by a medical entertainment company, and instead of keeping the data internal, we are sharing this information with the wider medical audience.

Identifiable individuals have granted consent for the use of their image in this publication.

### Dissemination and Marketing Methods

For this work, we disseminated the video on Facebook (Meta) because of its number of users and daily interactions, campaign optimizations, low cost of advertising, analytic capabilities, and sophisticated audience targeting that identifies specific audiences based on users’ interests and previous interactions [20]. DHN ran and coordinated the marketing campaign using their Facebook advertising account, and video performance data were captured using Facebook’s advertising management platform.

The video ran daily from September 7, 2021, to September 27, 2021. The video length was 5 minutes and 10 seconds long, and the production cost was US $4000. Projects with a similar method would cost significantly more to produce; however, costs were kept at a minimum by filming at three free locations and DHN’s in-kind production services, which included directing, editing, color correction, music and graphic licenses, and voice-over narration. The video consisted of two different campaigns. The purpose of the first campaign was to identify the optimal target audience who might be more engaged with the video content. For the first campaign, which was conducted from September 7, 2021, to September 10, 2021, we used Facebook targeting algorithms to disseminate the video to audiences who expressed interests in breast cancer research and to audiences who expressed interest in breast cancer organizations. We compared the engagement (number of views and outbound clicks to the S1501 clinical trial web page) between each group. The audience interested in breast cancer research had greater engagement and was chosen as our target audience for the second campaign, which ran from September 10, 2021, to September 27, 2021. Our second campaign disseminated the video to individuals who met the look-alike audience profile, which is an audience who shared similar indicated interests, characteristics, and behaviors to individuals who were interested in breast cancer research. This technique helped us identify potentially more engaged users who would more likely be interested in our video; this is where we spent the majority of our US $1000 advertising campaign budget.

### Measures and Analysis

To evaluate video engagement, we measured four outcomes:

- *Reach* was defined as the number of people exposed to the video during the advertisement campaign.
- *Retention* was defined as the length of time that individuals watched the video. We measured the number of individuals who watched for at least 3 seconds and at intervals of 25%, 50%, 75%, and 100%.
- *Outbound clicks* were defined as the number of clicks on a “learn more button” under the video that was linked to the SWOG Cancer Research Network’s S1501 patient information web page.
- *Outbound click-through rate* was defined as the percentage of times viewers saw a video and performed an outbound click.

We calculated frequencies and descriptive statistics to summarize each outcome.

## Results

Reach, retention, outbound clicks, and outbound click-through rate are summarized in Table 1.

**Table 1.** Educational video engagement outcomes for the S1501 study.

Outcomes	Value
Length	5 min 10 s
Video reach, n	61,456
Length of video watched, n (% retained)	
At least 3 seconds	47,566 (77.4)^a^
25%	226 (0.5)^b^
50%	84 (37.2)^c^
75%	44 (52.4)^d^
100%	34 (77.3)^e^
Outbound clicks, n	414
Percentage increase in visitors	41,300
Outbound click-through rate, n/N (%)	414/61,456 (0.67)

a N=61,456.

b n=47,566.

c n=266.

d n=84.

e n=44.

In our first campaign, we found that the group interested in breast cancer research performed better with video views and outbound clicks than the group interested in breast cancer organizations (9764 views and 54 outbound clicks vs 2513 views and 25 outbound clicks). This informed our target look-alike audience for the second campaign. The video had a total reach of 61,456 individuals. A total of 77.4% (47,566/61,456) watched at least 3 seconds and among those, 0.5% (226/47,566) watched 25% of the video. The number of viewers dropped at each consecutive retention interval; however, the retention rate increased at each consecutive retention interval past 25%. A month prior to launch, the S1501 patient information clinical trial web page had only 1 visitor. During the 21-day dissemination campaign period, the video received 414 outbound clicks from Facebook to the patient-facing clinical trial page. The outbound click-through rate was 0.67% (414/61,456).

## Discussion

### Principal Findings

We found that with an active digital marketing dissemination strategy and a very modest marketing budget (US $1000), the video substantially increased health information seeking behavior by increasing visitors to the SWOG’s S1501 patient information page from only 1 web page visit the month prior to campaign initiation to 414 web page visits during the campaign. Although research organizations have passively disseminated clinical trial–related content on social media platforms, this very active and intentional campaign to engage with the public is promising as it relates to clinical trial recruitment and illustrates the utility of using engaging digital content coupled with social media marketing as an effective strategy for clinical trial recruitment. Additionally, our study shows that digital and social media tools can be disseminated to specific target audiences at low cost, making it an accessible educational, recruitment, and retention strategy for clinical trials.

Our study shows that the health information seeking behavior of social media users may be impacted by immediate attrition. The average social media video watch time benchmark is 10 seconds, and Facebook’s best practices suggest that video advertisements be 15 seconds or less [9]. Considering that the average Facebook video watch time is 4.6 seconds [21], our video (5 min 10 s), which only retained 0.5% of 3-second viewers, illustrates the challenge of delivering important information in a succinct way. In addition to video length and the lack of engagement, another factor that may have contributed to this decline is that although we used Facebook algorithms to target individuals who were interested in breast cancer research and breast cancer organizations, it is plausible that only a subset of these individuals were interested in learning about clinical trials. Interestingly, viewership retention across other intervals was drastically higher (37.2% retention at the 50% interval, 52.4% retention at the 75% interval, and 77.3% retention at the 100% interval). Viewers who watched 25% of the video (1 min 17 s) tended to stay for the duration of the video, suggesting an engaged audience. Although a drop-off in retention is expected, our data suggest that the first 45-60 seconds may be the most instrumental in capturing audiences and maximizing engagement among viewers who are interested in the video topic area. It is noteworthy that the number of outbound clicks (n=414) was substantially higher than the number of people who watched the full video (n=34). This indicates that certain individuals were compelled to seek further health information before they finished watching the video, although we do not know when during the duration of the video that they performed an outbound click. These data show that *completely watching* the video was not necessarily correlated with seeking additional information (ie, outbound clicks) and further illustrates the importance of delivering compelling and engaging content within the first 60 seconds of video content. Finally, the outbound click-through rate (0.67%) was below the average outbound click-through rates of 0.89% across industries and 0.83% in health care, but above the 0.45% average in science [22,23], which may also provide insight into the level of engagement in the video.

In this burgeoning and ever-evolving social media landscape, digital marketing and its advanced analytical techniques allow researchers to engage with patients and potential trial participants where they are already consuming health information. It also allows for real-time data on what worked well and what did not work well, so that researchers can pivot their tact and capitalize on the most effective strategies to engage with an audience that is more likely to be interested in or eligible for their trials. Although this medium is novel in oncology, social media analytics have proven to be successful in informing engagement strategies for other industries, and future oncology research could benefit from these digital tools.

### Comparison to Prior Work

Our research is aligned with limited previous studies that found web-based infotainment videos to be an effective approach in increasing public understanding about science and health care among web-based health information seekers [14,24,25]. One study found that narrative infotainment videos compared to expository videos resulted in more likes by viewers without a university education and better information recall among viewers [14]. Study findings that assess study recruitment with and cost effectiveness of social media advertisement for study enrollment are mixed [26-29]. A recent scoping review reported that 9 (27%) out of 33 studies that used both social media and traditional methods for recruitment to clinical trials achieved or exceeded their enrollment target, and one study reported that social media outperformed other recruitment methods. The review did not specify how many of these studies used video as a recruitment method, highlighting the need for more research to understand if infotainment videos disseminated on social media to target audiences may be a significant strategy for facilitating clinical trial recruitment [24,30].

### Limitations

Our study is limited in that we were not the administrators of the S1501 study’s public facing web page and did not retain detailed information about visitor activities or characteristics (eg, location, frequency, and time of visit). This limited our ability to ascertain if each outbound click was from a unique or repeat visitor and our ability to understand additional characteristics that may have impacted health information seeking behaviors. We recommend that research teams be the administrators of both outgoing (where the advertisement played) and incoming (where visitors were sent) visitor analytics to maximize data utility. Additionally, our study did not test whether outbound clicks resulted in actual recruitment; therefore, we cannot make assertions that the increase in web page visitors contributed to study recruitment [31]. In addition, our video did not include subtitles in English nor any other language. Since 85% of Facebook users interact with the platform with the sound off [32], future digital media engagement studies should consider the use of language subtitles as this feature might facilitate engagement and accessibility with a larger audience. Another limitation is that our target audience indicators may not have been the most appropriate indicators to capture audiences interested in a specific clinical trial. We cannot ascertain that outbound clicks reached potential participants, as opposed to other individuals who may have been interested in clinical trials (eg, researchers and providers). More specialized or specific targeting (eg, those meeting study criteria) should be used to capture audiences who would most benefit from and be engaged by video media for specific clinical trials. Finally, we tested engagement using only one social media platform. Social media platforms vary in the average length of videos and average video watch times. It is plausible that social media platforms that are more conducive to longer viewing times (eg, YouTube) may have yielded different viewership and engagement outcomes.

### Future Directions

Emerging internet technologies and social media are widely used sources for health information. Our study found that infotainment disseminated using social media is a useful and effective approach in relaying complex health information, motivating interested viewers to seek additional health information, and driving public audiences to credible and reliable sources of information. It has promising utility in facilitating recruitment and retention strategies for cancer clinical trials and generating increased awareness about clinical trials among patients and the general public.

## Supplementary material

10.2196/56098 Multimedia Appendix 1 Southwest Oncology Group S1501 video.

## Abbreviations

DHN: Digital Health Networks
SWOG: Southwest Oncology Group
WHO: World Health Organization
